# New progress in zeolite synthesis and catalysis

**DOI:** 10.1093/nsr/nwac045

**Published:** 2022-03-09

**Authors:** Hao Xu, Peng Wu

**Affiliations:** Shanghai Key Laboratory of Green Chemistry and Chemical Processes, School of Chemistry and Molecular Engineering, East China Normal University, Shanghai 200062, China; Shanghai Key Laboratory of Green Chemistry and Chemical Processes, School of Chemistry and Molecular Engineering, East China Normal University, Shanghai 200062, China

**Keywords:** layered zeolite, germanosilicates, green synthesis, fast synthesis, bifunctional catalytic system, metal confined in zeolite

## Abstract

The rational design synthesis of zeolite catalysts with effective, environmentally benign and atom-economic routes is a major topic in the field of microporous materials, as it would avoid the high labor cost and inefficiency of traditional trial-and-error methods in developing new structures and dispel environmental concerns regarding the industrial mass production of zeolites. Catalytic applications of zeolite materials have expanded from conventional single functionalities, such as solid acids or selective oxidation catalysts to bi/multifunctionalities through combination with metals or metal oxides. This is a response to new requirements from petrochemical and fine chemical industries, such as precise control of product distribution, conversion of low-carbon resources for chemical production, and solutions to increasingly severe environmental problems related to CO_2_ and NO_x_. Thus, based on the systematic knowledge of zeolite chemistry and science that researchers have acquired in the past half-century and the development requirements, remarkable progress has been made in zeolite synthesis and catalysis in the past 10 years. This includes the manipulation of zeolitic monolayers derived from layered zeolites and germanosilicates to construct novel zeolite materials and effective and green zeolite syntheses as well as the synergistic interaction of zeolites and metal/metal oxides with different space distributions in the conversion of low-carbon resources. With many zeolite catalysts and catalytic processes being developed, our understanding of the close relationship between zeolite synthesis, structure and catalytic properties has deepened. Researchers are gradually approaching the goal of rationally designing zeolite catalysts with precisely controlled activity and selectivity for particular applications.

## Teaser

Remarkable progress has been made in both zeolite synthesis and catalysis in the past 10 years, including the manipulation of zeolitic monolayers derived from layered zeolites and germanosilicates to construct novel zeolite materials, the effective and green zeolite synthesis methods, the synergistic interaction of zeolite and metal/metal oxide with different space distributions in the conversion of low-carbon resources.

## INTRODUCTION

Zeolite, which has a crystalline structure and uniform pore channels, was first discovered in 1756. However, the scientific study of zeolites, including their practical application and artificial synthesis, started at the end of the 19th century. The zeolite family has since achieved significant expansion, with the number of topological structures increasing to 255 [1]. In addition, the diversity of framework-building atoms has expanded from the initial Si and Al atoms to Ti, Sn, Zr, Fe, P etc. [2,3]. The application of zeolite materials has changed from the field of adsorption and separation to that of heterogeneous catalysis.

The unique topology of the crystalline structure and subnano micropore channels endow zeolites with a molecular sieving ability and provide an intracrystal diffusion path and reaction space for the substrates, while their framework heteroatoms determine the catalytic functions. Even with the same kind of heteroatom, the catalytic ability could be comprehensively affected by changeable diffusion properties and different micro-environments [4]. The design synthesis of new zeolites will enrich the zeolite family and increase the possibility of constructing highly active microporous catalysts. The traditional research strategy was to synthesize a new zeolite via the blind trial-and-error method, resolve the structure and investigate the catalytic property. Inversely, considering the target of diffusion and catalytic functions, the strategy based on a rational design synthesis is more attractive and preferable.

The catalytic application of zeolites started in the 1960s with the use as solid acid catalysts in the cracking and refining of crude oil to produce transportation fuels and, thereafter, value-added chemicals via shape-selective catalysis [5]. The invention of the first titanosilicate (TS-1) expanded the application field to selective oxidation [6]. Subsequently, zeolites found application in environmental pollution, such as in the selective catalytic reduction deNO_x_ reaction and the catalytic removal of organic sulfur compounds [7,8]. As heterogeneous oxidation catalysts, zeolites offer significant advantages in constructing green catalytic systems, such as the epoxidation of propylene and cyclohexanone ammoximation [9]. However, the mass production of zeolite catalysts causes severe environmental problems, owing to the burning of organic structure-directing agents (OSDAs) and the discharge of wastewater during hydrothermal synthesis and postmodification [10]. Thus, an effective and green mass-production strategy is highly desired to realize the industrialization of zeolite catalysts, which provides the material foundation for related chemical processes.

With a shortage in oil resources, converting coal to chemicals through Fischer–Tropsch (FT) synthesis and activating low-carbon resources of CH_4_, CO_2_ and CH_3_OH to produce value-added hydrocarbons have become important supplementary routes and have been extensively studied in the past decade [11,12]. After a revolution in the petrochemical industry, zeolite found application in the construction of unique bifunctional catalytic systems in combination with metal/metal oxides [13]. These novel applications have inversely promoted the exploration of new synthetic strategies for zeolite–metal/metal oxide bifunctional catalysts [14].

Although discovered 2.5 centuries ago, studies on zeolites and the development of related techniques are still ongoing, benefiting from the mutual promotion between synthesis and catalysis. The main developments of the last decade, including the design synthesis of new zeolites via the manipulation of zeolitic monolayers, fast and green synthesis, catalytic application of zeolite in CO and CO_2_ conversion, and synthesis and catalytic application of zeolite-confined metal catalysts, are reviewed herein. The challenges and limitations of these syntheses and applications are also discussed.

## SYNTHESIS OF ZEOLITES VIA THE MANIPULATION OF ZEOLITIC MONOLAYERS

The use of novel OSDAs with special molecular configurations and suitable dimensions and functional groups, under appropriate synthetic conditions achieved by tuning the alkalinity, water/silica ratio, heteroatoms and crystallization temperature, has contributed to the discovery of a variety of zeolites [15]. The novel ZEO-1 zeolite is a recent example, and was synthesized using a new quaternary phosphonium as the OSDA. It possesses intersecting 3-dimensional (3D) extralarge pores and can compete with the USY zeolite in the catalytic cracking reaction [16]. This direct synthetic strategy still relies on the trial-and-error method because of an inadequate understanding of the crystallization mechanism related to the inorganic–organic and/or host–guest chemical interactions at the molecular level. In extremely limited cases, the direct hydrothermal synthesis of zeolites for a target catalytic reaction can be rationally designed by choosing an OSDA that mimics the intermediate molecules in the reaction [17,18]. In contrast, the fabrication of zeolitic monolayers derived from layered zeolite precursors or quasi-layered materials of germanosilicates, is a more rational method for constructing zeolite structures.

### Synthesis and structural modification of layered zeolites

**Figure 1.**
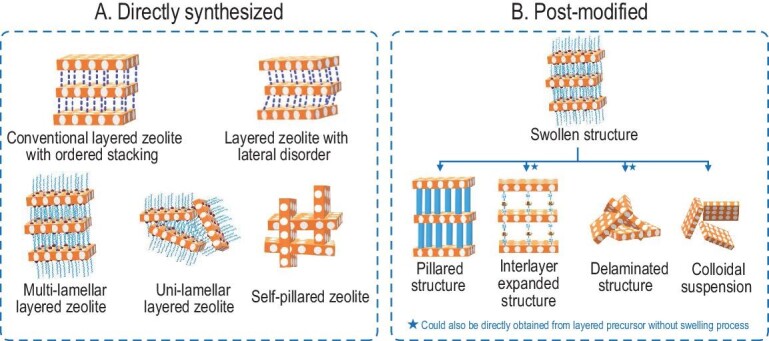
Structural diversity of layered zeolites.

Hydrothermally synthesized layered zeolitic precursors possess a unique structure that expands within only two dimensions, while the third dimension is interrupted by OSDAs, resulting in a weak hydrogen bonding linkage between the neighboring zeolitic layers. By changing the stacking style of monolayers within layered zeolites, novel structures with high external surface area or interlayer-expanded pores can be rationally designed (Fig. 1). The most widely used postmodifications are phase delamination or exfoliation, interlayer silylation and pillaring. The delamination process offers randomly stacked layers with a large external surface [19]. Silylation with organic silanes constructs enlarged interlayer pores and expanded structures via the introduction of additional Si atoms. Silylation agents range from monomeric, dimeric and 4-member ring types to bulky types containing phenyl groups [20–22]. The use of amorphous silica or metal oxide as pillars creates interlayer mesopores, resulting in microporous and mesoporous hybrids [23,24]. Taking the most studied MWW layered zeolite as an example, over 17 kinds of derivative structures have been synthesized through postmodifications or direct syntheses, and they exhibit superior catalytic performance compared to the traditional 3D MWW structure [25]. Inspired by the structural diversity of layered zeolites and their potential catalytic applications, the development of layered zeolitic precursors and the manipulation of the stacking style of zeolitic layers has significantly improved in the past ∼10 years.

Traditional layered zeolites are mainly spontaneously synthesized by hydrothermal synthesis and no particular formation mechanism has been proposed. A breakthrough was made by Ryoo *et al.*, reporting the rational design synthesis of lamellar MFI zeolite, which had never been previously synthesized as a lamellar precursor [26]. This achievement was realized using a bifunctional OSDA, with the hydrophilic ammonium cation groups directing the crystallization of MFI zeolitic layers and the hydrophobic long alkyl chain interrupting the rigid connection along the *b* axis. Benefiting from the large interlayer space, the removal of the OSDA by calcination created a meso/microporous structure, which was effective in depressing the coke formation for the methanol-to-gasoline reaction as well as processing the bulky substrates. This sandwich-like MFI layered structure with an alternative arrangement of an organic OSDA layer and inorganic silicate or aluminosilicate layer can be further modified to provide a pillared or delaminated structure [27–29]. Although the as-made sandwich-like MFI structure resembles the swollen structure of traditional lamellar zeolites, part of the interlayer OSDA molecules are firmly fixed in the intralayer pore channels, which prohibits direct silylation in an acidic medium. Liu *et al.* intercalated an organic silane into the interlayer space of an MFI layered structure after removing the occluded OSDA via an acid treatment and successive photochemical extraction [28]. By modifying the interlayer silane with ammonium groups, the interlayer-expanded MFI zeolite served as an acid–base bifunctional catalyst. With the OSDA firmly fixed in the intralayer structure, the as-made lamellar MFI can serve as an acid–base bifunctional catalyst, with the OH^−^ groups compensating for the exposed ammonium groups as the base sites, and the intralayer Al atoms as the acid sites [30]. In contrast, the exfoliation of the layered MFI zeolite assisted by polystyrene was directly performed over the as-made structure [27]. The pillaring of the layered MFI with amorphous silica or the combination of silica and metal oxide can also be readily performed over the as-made structure [29].

By introducing aromatic groups into bifunctional OSDAs, Che *et al.* synthesized a series of lamellar MFI zeolites with unique mesostructures that formed because of the strong π–π stacking and geometrical matching of the aromatic groups and MFI structure [31]. This synthetic strategy using bifunctional OSDAs has been extended to other traditional 3D rigid zeolites, including ^*^MRE [32], MTW [26] and AlPO [33]. Nevertheless, no further postmodifications have been reported for these novel layered zeolites. For the existing MWW lamellar zeolite, the use of bifunctional OSDA directly produced delaminated structures, with the disordered stacking of unilamellar MWW nanosheets [34,35]. In the synthetic system of ^*^BEA, CHA and MOR zeolites, the use of bifunctional OSDAs only resulted in nanocrystals, such as nanosheets or nanosponges, which exhibited superior catalytic performance compared to traditional bulk crystals due to a reduced diffusion path or specific exposed active sites [36–38]. As a special case, when a bifunctional OSDA was introduced into the synthetic system of a nonporous NON zeolite, planer defects were created, resulting in two derivative NON structures with short-range 8R pores and enlarged inner cages [39]. The delicate design of a bifunctional OSDA, such as the structure of the ammonium head and the length between two ammonium cations, is critical for the successful synthesis of a real layered zeolite as it controls the interaction and structural matching between an inorganic zeolitic framework and organic OSDAs. However, the complex and expensive synthesis of these Gemini-type OSDAs is challenging, despite the novel layered or defective structures with unique catalytic performance. Additionally, preserving the large surface area and pore volume in layered zeolites is challenging during the shaping process to obtain an industrial catalyst. This has hindered the industrialization of these active layered zeolites.

Progress has been achieved in the development of modification techniques, mainly in the simplification of traditional complex or structure-corrosion procedures. The swelling process, with the long-chain surfactant intercalating into a layered structure, is necessary to create sufficient interlayer space before delamination, pillaring and interlayer expansion with bulky silanes. However, the traditional swelling process performed in an alkaline medium easily causes structural dissolution, possibly leading to the formation of mesoporous silica as an impurity phase [40]. Direct delamination and interlayer expansion from lamellar zeolite precursors without a preswelling process have been extensively studied. Katz *et al.* proposed a mild delamination process for MWW and FER-type lamellar zeolites to produce the phase-delaminated materials UCB-1 and UCB-2, with F^−^ and Cl^−^ ions selectively attacking the framework Si and Al atoms, respectively [41,42]. The high product yield and absence of Q^2^ groups confirmed the well-preserved layer structure in this mild delamination process. Starting from a particular B-containing MWW lamellar zeolite precursor (ERB-1), a simple strategy, involving the isomorphous substitution of B by Al atoms, was proposed by Katz *et al.* [43]. The use of a neutral OSDA in synthesizing a B-MWW lamellar precursor and the precise control of the treatment temperature were critical for the formation of a delaminated structure [43]. In addition to delamination, the isomorphous substitution introduced Al-related acid active sites. After removing excess Al atoms, the 2-methoxynaphthalene conversion over the phase-delaminated material of ERB-1-del was more than twice that of the corresponding 3D structure in the Friedel–Crafts acylation of 2-methoxynaphthalene with acetic anhydride. However, this strategy was restricted by the lamellar precursor and was difficult to generalize. The direct synthesis of a swollen structure is an alternative method for avoiding the traditional structure-corrosion procedures. Wu *et al.* synthesized the swollen MWW structure ECNU-7P by introducing a long-chain surfactant into the secondary crystallization process of ITQ-1, where the simultaneous incorporation of Al or Ti active sites was possible [44,45]. The direct calcination of ECNU-7P resulted in a hierarchical MWW structure, exhibiting higher activity in processing bulky substrates (1,3,5-triisopropylbenzene and cumene hydroperoxide) compared to the conventional 3D MWW structure. As for the interlayer silylation with bulky silanes, Wu *et al.* inserted a bulky single-four-ring (*s4r*) silane into the interlayer space of the PLS-3 lamellar zeolite and constructed an extralarge 14 × 12R pore channel via deconstruction–reconstruction, avoiding the traditional structure-corrosive swelling process [20]. The partial removal of the original OSDA, tetraethyl ammonium (TEA^+^), in the interlayer space led to the formation of a subzeolite, ECNU-8, with disordered layer stacking. This was reconstructed in the presence of a bulky OSDA, 4-amino-2,2,6,6-tetramentylniperidine, to produce a lamellar precursor with enlarged interlayer space, favoring silylation. The layered zeolite-derived catalysts with limited unit cells or interlayer-expanded pores significantly released the diffusion constraints that traditional microporous zeolites suffer and exhibited superior activity in many reactions, which were detailed in a previous review [46].

### Structural diversity of germanosilicates as potential layered zeolites

Germanosilicates are unique heteroatom-containing zeolites, known by large or extralarge pore channels and novel topologies. The more flexible Ge–O bond, compared with the Si–O bond, favors the formation of small building units, such as the double-4-ring (*d4r*) and *d3r*, which facilitates the construction of large or extralarge pore structures, e.g. ITQ-37 [47], IM-12 [48] and CIT-13 [49]. Despite the open porosity-derived significant potential in processing bulky substrates, germanosilicates suffer serious obstacles in practical application, owing to their extremely low hydrothermal stability. Ge–O bonds tend to hydrolyze under humid conditions, even at room temperature [50]. Nevertheless, the instability endows germanosilicates with structurally modifiable properties and diversity.

**Figure 2.**
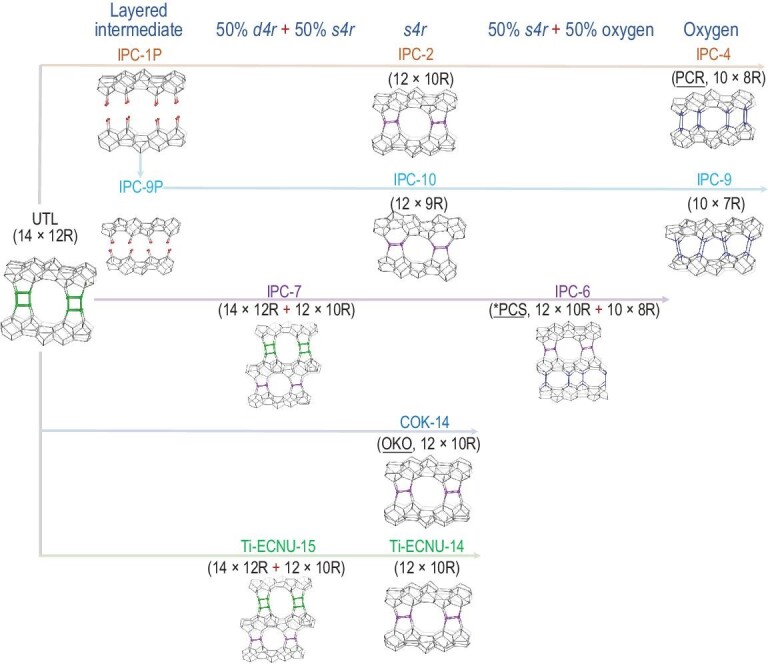
Structural transformations of UTL-type germanosilicate.

Cejka *et al.* selectively removed the Ge-rich *d4r* units in a UTL-type IM-12 zeolite via acid treatment, producing a layered material, IPC-1P, which confirmed that germanosilicates can be used as potential layered zeolites, initiating structural diversity for germanosilicates (Fig. 2) [51]. A novel zeolite, IPC-4, with intersecting 10 × 8R pores, was obtained through the organization of monolayers in IPC-1P with octylamine and subsequent calcination. The IPC-4 structure has been recognized by the International Zeolite Association (IZA) with the code ‘PCR’ [52]. By organizing the monolayers with a silylation agent, diethoxydimethylsilane (DEDMS), and subsequent calcination, another novel zeolite, IPC-2, was produced with interlayer 12 × 10R pores. The aforementioned structural transformation, including the initial structural assembly to obtain the parent germanosilicate, disassembly in an acidic medium to a layered intermediate, organization with organic amine or silane, and reassembly process via calcination, was defined as the ADOR (Assembly–Disassembly–Organization–Reassembly) strategy, which is particularly useful in the postsynthesis of new zeolites from germanosilicates [53]. The alternative arrangement of a stable Si-rich layer and unstable Ge-rich layer in the IM-12 zeolite was critical for an ADOR structural transformation. IPC-2 and IPC-4 possess the same layered structure as their parent IM-12 zeolite but exhibit different interlayer linkages, changing from the *d4r* to *s4r* units in IPC-2 and oxygen in IPC-4. The change in the interlayer linkage induced variation in the relative position along the layer stacking direction. The relative position of the two neighboring layers perpendicular to the layer stacking direction can be tailored by treating IPC-1P with a choline hydroxide solution, yielding the intermediate, IPC-9P [54]. The direct calcination of IPC-9P resulted in a new zeolite, IPC-9, with an odd-number ring pore (10 × 7R), while the silylation of IPC-9P with DEDMS produced an interlayer-expanded structure, IPC-10, with 12 × 9R pore channels. The interlayer linkage in the aforementioned IPC-*n* (*n* = 2, 4, 9 and 10) samples was uniform. Two different linkages could appear in the same structure. Starting directly from the IM-12 germanosilicate, two other novel zeolites, IPC-6 and IPC-7, with the regular distribution of two different interlayer linkages in one structure, were obtained by carefully controlling the acid concentration and treatment time, which affected the speed of a Ge removal-induced interlayer structural collapse and an Si species-assisted structural repair [55]. The interlayer linkage was 50% *s4r* and 50% oxygen for IPC-6, while IPC-7 possessed 50% *d4r* and 50% *s4r*. The IZA has recognized IPC-6 with the code ‘^*^PCS’. By changing the acid treatment time of the IM-12 zeolite at an extremely high temperature of 463 K, Wu *et al.* achieved the continuous tailoring of interlayer pore dimensions and obtained four daughter structures analogous to IPC-*n* (*n* = 2, 4, 6 and 7) [56]. Kirschhock *et al.* proposed an inverse sigma transformation of IM-12 with a highly concentrated HCl solution (12 M) at 368 K, producing a daughter structure, COK-14, analogous to IPC-2 [57]. The structure of COK-14 has been recognized by IZA as OKO. In addition to the acidic medium, the Ge removal-derived structural transformation can be realized in a reduction atmosphere. By reducing the framework Ge atoms into extraframework Ge clusters in an H_2_ atmosphere and controlling the treatment time, Wu *et al.* prepared two daughter structures analogous to IPC-2 and IPC-6 [58].

**Figure 3.**
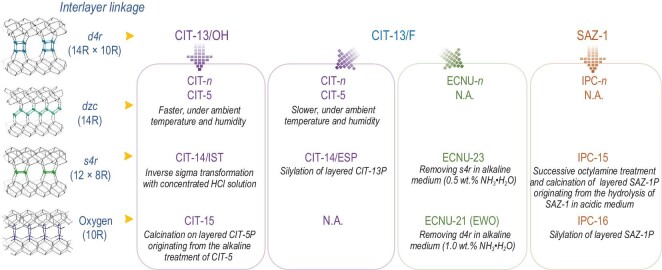
Source and treatment-condition-dependent structural transformations of ^*^CTH-type germanosilicate.

The ADOR strategy has been extended to other germanosilicates, e.g. UOV-, IWW- and ^*^CTH-type zeolites [50,59,60]. Among them, the structural transformation of ^*^CTH-type germanosilicates is the most extensively studied, and its number of daughter structures is higher than that of UOV- and IWW-type germanosilicates (Fig. 3). The CIT-13 germanosilicate obtained in the F^−^ containing synthetic system is the first zeolite reported to exhibit the ^*^CTH topology, comprising *cfi*-layers pillared by interlayer *d4r* units, which were located randomly, owing to the presence of two crystallographically equivalent positions on the *cfi*-layers [49]. However, the structural transformation of the ^*^CTH-type germanosilicate was reported for the first time over the SAZ-1 germanosilicate analogous to the CIT-13 zeolite [61]. With the same ADOR strategy reported in dealing with the UTL-type germanosilicate, the hydrolysis of the SAZ-1 zeolite in an acidic medium produced a layered intermediate of SAZ-1P, which was organized by octylamine and reassembled by calcination to produce a daughter structure, IPC-15, with interlayer oxygen linkage. The silylation of the SAZ-1P layered intermediate resulted in another daughter structure, IPC-16, with interlayer *s4r* units. Although high-resolution transmission electron microscopy images confirmed the formation of 12 × 8R pore channels in IPC-16 and one-dimensional (1D) 10R pore channels in IPC-15, the X-ray diffraction patterns of IPC-15 and 16 zeolites were poor. Using the CIT-13 germanosilicate as a starting material, Wu *et al.* found it difficult to obtain daughter structures in an acidic medium because of too many stable Si–O–Si bonds in the interlayer *d4r* units, as revealed by the ^19^F magic-angle spinning nuclear magnetic resonance (MAS NMR) spectra [62]. Thereafter, a mild alkaline treatment was proposed to completely remove the interlayer *d4r* units by cleaving the Si–O and Ge–O bonds, and an ECNU-21 zeolite (1D, 10R) with a highly ordered structure was obtained, which was analogous to IPC-15 and recognized by the IZA with the code ‘EWO’. What is noteworthy is that the polymorph stacking phenomenon in the pristine ^*^CTH-type germanosilicate disappeared because of the complete removal of interlayer *d4r* units, resulting in an ECNU-21 with a pure crystalline structure. By reducing the alkaline concentration, only *s4r* units were removed to produce an ECNU-23 zeolite with interlayer 12 × 8R pores [63]. Davis *et al.*, the first research group to report the CIT-13 zeolite, unexpectedly observed an extremely slow structural transformation (>100 days) from the CIT-13 to CIT-5 zeolite spontaneously at ambient temperature and humidity [50]. This structural transformation was caused by a change in the interlayer linkage from *d4r* units to double-zigzag chains (*dzc*) upon the hydrolysis of partial Ge–O bonds. This benefited from the two crystallographically equivalent positions on the *cfi*-layers. No similar structural transformation has been observed for other germanosilicates. The high Ge content in the parent CIT-13 zeolite can accelerate the structural transformation process. A fast structural transformation from the CIT-13 to CIT-5 zeolite can be realized by synthesizing the parent CIT-13 zeolite under F^−^-free conditions (designated CIT-13/OH), where the Ge atoms tend to be located in proximal T-sites in the *d4r* units [64]. With a comparable Si/Ge ratio of ∼4.3 but different synthetic media, the CIT-13 zeolite synthesized with F^−^ ions (designated CIT-13/F) took 85 days to complete the structural transformation to CIT-5, while CIT-13/OH only required 12 days. The aggregated Ge distribution in the *d4r* units allowed the inverse sigma transformation from CIT-13/OH to a daughter structure, CIT-14, analogous to IPC-15 and ECNU-23. By treating the CIT-5 zeolite derived from CIT-13/OH with an alkaline solution, Davis *et al.* obtained a layered intermediate, CIT-5P, which was transformed into the CIT-15 zeolite upon calcination with a structure analogous to IPC-16 and ECNU-21. Comparing the structural transformation of IM-12, SAZ-1, CIT-13/F and CIT-13/OH, the Ge content and distribution in the interlayer *d4r* units were extremely critical in manipulating the germanosilicate structures, in addition to the alternative arrangement of a stable Si-rich layer and instable Ge-rich layer.

Furthermore, the Si-rich intralayer structure can affect the structural transformation. Dissimilarly to the nonporous dense layer structures in the UTL and ^*^CTH zeolites, the Si-rich layers in the IWW germanosilicate contain pores with low framework density. Thus, the typical acid treatment in an aqueous solution easily caused structural deconstruction, hindering the production of highly crystalline daughter structures [65]. Cejka *et al.* developed a vapor-phase transport strategy for the structural transformation of an IWW structure to a daughter structure, IPC-18, with interlayer *s4r* linkage [60]. Wu *et al.* synthesized an IWW zeolite with an extremely low Si/Ge ratio of 1.2 and achieved a structural transformation from IWW to IPC-18 in pure water at 298 K, which excellently preserved the intralayer structures [66].

Compared to the structural transformation of germanosilicates, studies on catalytic application are limited. Wu *et al.* observed that the germanosilicates were active in the Baeyer–Villiger oxidation reaction with the framework Ge atoms as active sites. However, highly concentrated H_2_O_2_ (>50 wt%) was required to decrease the amount of H_2_O in the reaction system and prohibit the structural degradation of germanosilicates [67]. The stability of germanosilicates could be significantly enhanced by removing the Ge atoms. By removing the Ge-rich *d4r* units from the CIT-13 zeolite, Wu *et al.* synthesized a high-silica ECNU-21 zeolite with a Si/Ge ratio of 33. The residual Ge atoms in the framework, exhibiting Lewis acidity, can catalyze the hydration reaction of ethylene oxide to produce ethylene glycol with a greatly reduced amount of water [62]. Under relatively mild reaction conditions compared to the industrial process, ECNU-21 achieved >96% ethylene oxide conversion and ethylene glycol selectivity after 28 h, and the catalytic performance was well-maintained after at least six reuse and regeneration cycles. More studies focused on the Ti- or Sn-containing germanosilicates and their derived structures. Cejka *et al.* introduced Ti active sites into the UTL-type zeolite via direct hydrothermal synthesis and hydrolyzed the Ti-UTL zeolite to produce a layered intermediate, Ti-IPC-1P. Subsequently, structural reorganization and reassembly were performed over Ti-IPC-1P to obtain interlayer pillared Ti-IPC-1PI and Ti-IPC-2 [68,69]. The three titanosilicates with identical intralayer structure but different interlayer pore sizes showed that the catalytic activity depended on the pore size in the epoxidation reaction of olefins and the selective oxidation of bulky sulfides, with a typical turnover number (TON) in the order of Ti-IPC-1PI >> Ti-UTL > Ti-IPC-2 [68,69]. However, the active sites in the parent UTL germanosilicate suffered severe leaching in poststructural modifications involving acid treatment. Introducing the active sites in or after the structural transformation procedure was more practical. A highly stable Sn-UTL zeolite was synthesized by Wu *et al.*, by introducing Sn atoms in the structural stabilization process, where a fast deconstruction–reconstruction occurred [70]. Sn-UTL with extralarge 14 × 12R pores exhibited higher activity in the Baeyer–Villiger oxidation reaction, compared to Sn-Beta zeolites. A similar strategy was applied to synthesize a Ti-IWV zeolite using the 3D-2D-3D structural transformation, where the Ti insertion and structural reconstruction process of a layered intermediate derived from a Ge-rich IWV zeolite were simultaneously realized [71]. Ti-IWV showed extremely high activity in the epoxidation of cyclooctene, with a TON value >10 times higher than other traditional titanosilicates, owing to the special adsorption capacity of the extralarge 14R inner cavity in the IWV structure [71]. Wu *et al.* attempted to introduce active sites after the structural transformation via a solid–liquid reaction. Treating the layered precursor of ECNU-14 and ECNU-15 with an H_2_TiF_6_ aqueous solution inserted the Ti active sites and made the structure more ordered [56]. In the epoxidation reaction of cyclohexene with bulky *tert*-butyl hydroperoxide, the catalytic activity of the three titanosilicates was pore-size-dependent in the order of Ti-UTL > Ti-ECNU-15 > Ti-ECNU-14.

The structural transformation of germanosilicates via the ADOR strategy is effective in synthesizing zeolites, something which is not feasible via direct hydrothermal synthesis. Additionally, the ADOR strategy can enhance structural stability by removing partial Ge atoms. However, the waste of expensive Ge atoms in the structural transformation and stabilization process constitutes a problem. The recovery and recycling of Ge atoms have been confirmed to be possible. Cejka *et al.* recovered 78%–94% of Ge atoms from IWW, ITH and UTL zeolites. The recovered Ge atoms were used to successfully synthesize germanosilicates [72]. The recycling of Ge atoms from the UWY germanosilicate in the structural stabilization process has been reported by Wu *et al.* [73]. Thus, ADOR is a sustainable route for synthesizing zeolites with high stability for potential catalytic applications.

## ADVANCED SYNTHETIC STRATEGIES FOR ZEOLITES WITH FAST AND ENVIRONMENTALLY BENIGN CRYSTALLIZATION

Although 255 zeolites have been recognized by the IZA, the number of zeolites with real industrial application is limited (<20). Many zeolites have promising applications in industry, but the main difficulties are the long crystallization time, use of costly OSDAs and environmental burden related to the disposal of OSDA waste. Thus, novel synthetic strategies, including the hydroxyl free radical-assisted, OSDA-free and solvent-free methods, have been extensively studied in the past decade.

### Hydroxyl free radical-assisted synthesis

**Figure 4.**
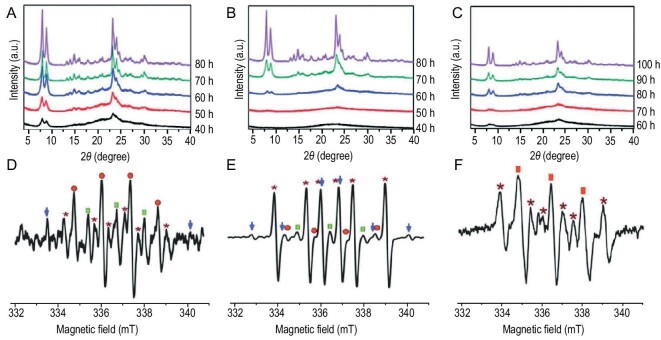
Acceleration processes using Fenton's reagent. (A--C) Crystallization processes of silicalite-1 at 343 K under (A) Fenton conditions, (B) ultraviolet (UV) conditions (4.0 mW/cm^2^) and (C) dark conditions. (D--F) Electron paramagnetic resonance (EPR) spectra of the TPAOH–TEOS–H_2_O system under (D) Fenton conditions, (E) UV conditions (4.0 mW/cm^2^) and (F) dark conditions. The EPR signals are marked by the following: red circles, •OH; green rectangles, oxidized DMPO radicals; blue arrows, silicon-based radicals; asterisks, ethanol radicals; and red rectangles, oxidized BMPO radicals. Reprinted with permission from ref. [75].

The acceleration of zeolite crystallization via microwave heating is a hot subject in zeolite synthesis [74]. However, it rapidly declined because of difficulties with industrialization. Recently, Yu *et al.* reported that the hydroxyl free radicals provided by UV irradiation or Fenton's reagent shortened the crystallization time of zeolites by boosting the nucleation stage, although they hardly affected the crystal growth stage [75]. The existence of hydroxyl free radicals in the synthetic system was confirmed by electron paramagnetic resonance (EPR) experiments with a spin-trapping agent (Fig. 4), and the density functional theory calculations confirmed that the OH· in the synthetic gel increased the rate of depolymerization and repolymerization by decreasing the activation energies. This strategy worked for numerous zeolites, including Na-A, Na-X, NaZ-21 and S-1. In the crystallization of aluminosilicates, OH· was confirmed to favor the formation of Si–O–Si bonds rather than Si–O–Al bonds. Benefiting from this, Yu *et al.* synthesized a high-silica Y zeolite (Si/Al = 6.35) under OSDA-free conditions, surpassing the Si/Al ratio limit in previous reports [76]. In addition to UV irradiation, Chen *et al.* used Gamma-ray irradiation to provide OH· and accelerated the crystallization of NaA, NaY, S-1 and ZSM-5 zeolites [77]. Gamma rays can penetrate a stainless-steel autoclave to reach water in the synthetic mixture, which was impossible during UV irradiation. In addition to the direct zeolite synthesis, the poststructural stabilization of germanosilicates can be achieved under the extremely mild conditions of room temperature and neutral media via the hydroxyl free radical route, which accelerated the breaking of Si–O–Si bonds and the subsequent substitution of Si for framework Ge [78].

### Solvent-free synthesis strategy

Traditionally, zeolites are crystallized under solvothermal conditions with a large amount of water or organic solvent. Water is used in most cases, which inevitably raises the environmental concern of polluted water. In addition, the high pressure created by water at elevated temperatures constitutes safety problems in large-scale production. Early in the 1990s, researchers crystallized amorphous dry gel into a ZSM-5 zeolite under NH_4_F vapor using the vapor-phase transport method, indicating that the presence of water in zeolite synthesis was unnecessary [79]. However, it was observed that water can be produced during crystallization. Recently, Xiao *et al.* proposed that a trace amount of water from anhydrous starting materials, serving as the ‘catalyst’, was sufficient for crystallization [10,80]. The solvent-free synthetic strategy, which involves simply grinding the solid starting materials, as well as subsequent heating, was successful for at least 20 kinds of zeolites. In addition to the trace amount of water, F^−^ ions can function as a catalyst for depolymerization and repolymerization [81]. In the special case of silicoaluminophosphate synthesis, even a trace amount of water was not required because the interaction between the raw materials could produce water as a by-product [82].

**Figure 5.**
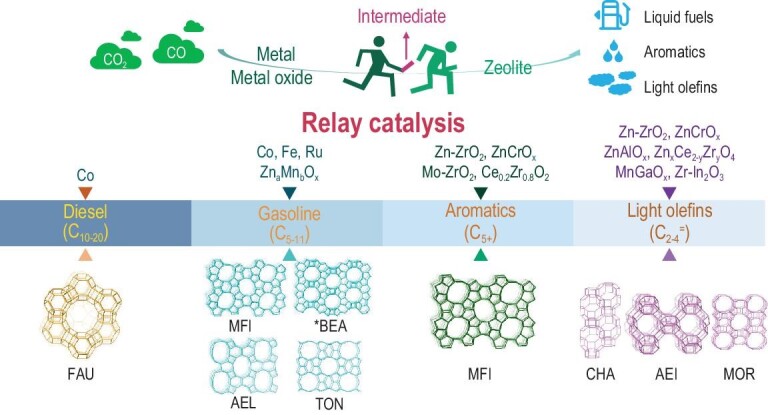
Metal (metal oxide)/zeolite composites as bifunctional or multifunctional catalysts for the conversion of CO and CO_2_.

Without the problem of the high pressure created by water, the crystallization temperature of a solvent-free system can be enhanced (>200°C) to accelerate the crystallization speed, and results in a higher space-time yield (STY) compared to traditional hydrothermal synthesis [83]. Furthermore, the solvent-free synthetic system provided a near-perfect environment for deep investigation of the crystallization mechanism using NMR and Raman spectra. This was because the limited amount of water prevented the interference of complex intermediates formed in the liquid phase [84,85]. Notably, mesopores were formed in SAPO-34 and ZSM-5 synthesized under solvent-free conditions without the addition of any mesoporogen agent, and the mesopore size increased with an increase in the crystallization time (Fig. 5) [82,86]. It was suggested that these mesopores were caused by the imprint of gaseous expansion, but no solid proof has been provided. In addition, the morphology can be tailored in solvent-free synthesis by adding a surfactant [87]. The surfactant molecules were selectively adsorbed on the crystal surface and prohibited the continuous growth of a specific plane. In contrast, the surfactant molecules behaved differently in a hydrothermal synthetic system and tended to form micelles inside the silica matrix.

Solvent-free synthesis can be further combined with the OSDA-free strategy to synthesize ZSM-5 and Beta zeolites, providing a green and inexpensive route for the two zeolites that has important industrial application [85]. It eliminates environmental and economic concerns and is promising for large-scale industrial application, which is the ultimate pursuit of zeolite synthesis. However, the extension of the solvent-free strategy to more zeolites is still challenging since most OSDAs are in hydroxide form, hardly directing the crystallization in solid salt form. Recently, the solvent-free strategy has been used to prepare zeolite-confined metal/metal oxide catalysts [88–90], which will be discussed in the section of ‘SYNTHESIS AND CATALYTIC APPLICATIONS OF METAL@ZEOLITE CATALYSTS’.

## ZEOLITE AS THE SHAPE-SELECTIVE ACID CATALYST COMPONENT IN THE CONVERSION OF CO AND CO_2_ TO HYDROCARBONS

Zeolites, as shape-selective solid acid catalysts, have caused a revolution in the field of C1 chemistry in the past five years, by cooperating with metals, metal carbides or metal oxides to break the limitation of the Anderson–Schulz–Flory (ASF) distribution in traditional FT synthesis. The metal, metal carbide or metal oxide component transforms the syngas or CO_2_ into intermediates, such as heavy hydrocarbons, olefins, methanol/dimethylether (DME) and ketene, while the zeolite component is responsible for the selective C–C cleavage, isomerization and C–C bonding catalyzed by the acid sites, constructing a highly selective bifunctional catalytic system for the synthesis of liquid fuels, aromatics or light olefins (C_2–4_^=^ ) [11,12,91]. The product selectivity is mainly dependent on the pore structure of the zeolite, although the acidity, the size of metal/metal carbide/metal oxide and spatial distribution of the two components can affect the product distribution (Fig. 5).

The direct synthesis of diesel-range hydrocarbons (C_10–20_) with a selectivity of 60% from syngas has been achieved using the bifunctional catalyst of Na-form mesoporous Y zeolite-supported Co metal [92], with a CO conversion of 40% at 503 K, breaking the selectivity limitation of 39% according to conventional ASF distribution. Owing to the absence of Brønsted acid sites, the heavy hydrocarbons formed on the Co metal component were supposed to undergo hydrogenolysis rather than hydrocracking over the Na-form mesoporous Y zeolite component. By changing the metal cations in the mesoporous Y zeolite, the product distribution was altered. Replacing Na with Ce, La and K cations resulted in a C_5–11_, C_8–16_ and C_10–20_ selectivity of 74%, 72% and 58%, respectively [93]. Additionally, the mean size of mesopores in the Na-formed Y zeolite affected the selectivity of C_10–20_, with the optimized mesopore size at ∼15 nm [92].

To shift the product distribution to the gasoline range (C_5–11_), the H-ZSM-5 zeolite was adopted to cooperate with the metal nanoparticles (Co, Fe or Ru) and catalyze the hydrocracking and isomerization of heavy hydrocarbons over Brønsted acid sites. H-ZSM-5 showed higher C_5–11_ selectivity than the H-MOR and HY zeolites, owing to its stronger Brønsted acidity [94]. However, the diffusion constraints in the microporous ZSM-5 zeolite easily cause over-cracking and carbon deposition. Introducing mesopores (∼5 nm) into the ZSM-5 component of the Ru/ZSM-5 catalyst increased C_5–11_ selectivity from 47% to 79% and significantly suppressed the over-cracking with decreased CH_4_ and C_2–4_ selectivities [95]. A special mesoporous ZSM-5 zeolite with nanosponge morphology, synthesized with the Gemini-type OSDA, was used to support Co metal, and the obtained bifunctional catalyst exhibited a high C_5–11_ selectivity of 73.8% at 493 K, which was considerably higher than the bulk ZSM-5 zeolite with a selectivity of 40.3% [96]. Notably, the proportion of olefins and *i*-paraffins was larger than that of *n*-paraffins in the gasoline-range products because the ZSM-5 nanosponge provided an extremely short diffusion length for the branched hydrocarbons. In addition to the most used ZSM-5 zeolite in the direct synthesis of gasoline from syngas, mesoporous Beta (meso-Beta) zeolite was once reported to cooperate with Ru metal, providing a C_5–11_ selectivity of 77% [97]. In contrast to the effective H-ZSM-5 with Brønsted acidity, the meso-Beta, obtained by NaOH treatment-induced desilication without further NH_4_^+^-exchange, exhibited Lewis acidity and demonstrated higher C_5–11_ selectivity than that of the H-type meso-Beta [97]. For the direct synthesis of C_5–11_, Bao *et al.* proposed the oxide-zeolite (OX-ZEO) concept using Zn_a_Mn_b_O_x_ and the shape-selective 1D zeolite of SAPO-11 and ZSM-22 as the catalyst [98]. The C_5–11_ selectivity could be as high as 76.7%, with an outstanding *iso*/*n*-paraffin ratio of 15 [98]. Although the exact intermediate species have not been identified, the authors indicated the possibility of methanol and ketene.

**Figure 6.**
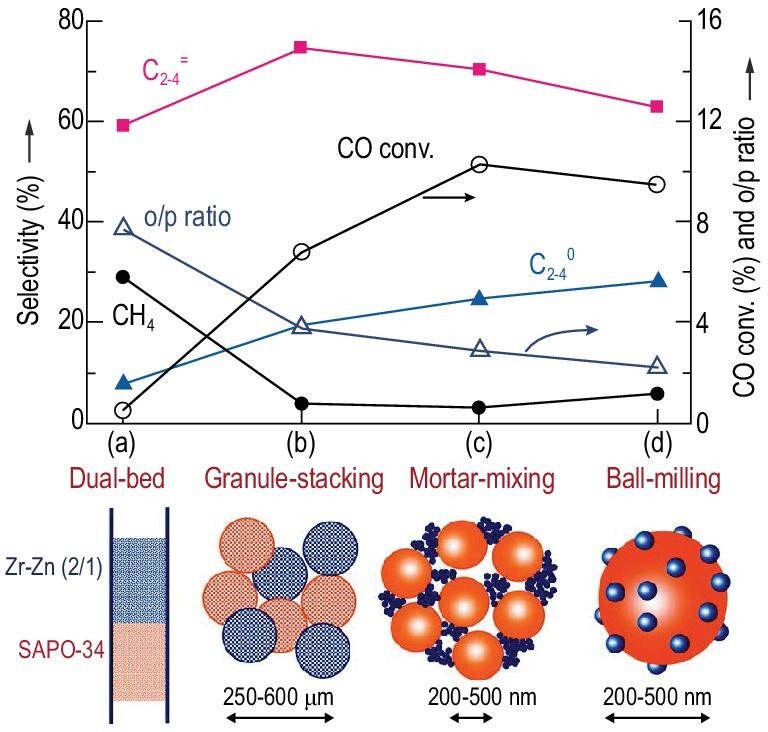
Effect of integration manner on the catalytic behaviors of the composite catalysts containing Zr–Zn (Zr/Zn = 2 : 1) oxide and SAPO-34 (24 h). (a) Dual-bed configuration. (b) Stacking of granules with sizes in the range of 250–600 μm. (c) Simple mixing of the two components in an agate mortar. (d) Ball-milling of the components together for 24 h. o/p ratio denotes the C_2_–C_4_ olefin/paraffin ratio. Reprinted with permission from ref. [99].

Light olefins, the most important building blocks for bulk chemicals, can be directly synthesized from syngas with the bifunctional catalyst of the CHA-type zeolite (SAPO-34 and SSZ-13) and metal oxide. The pore size of CHA-type zeolite correlates with the molecular size of light olefins. With a small amount of ZnO dissolved in the matrix of ZrO_2_ as the metal oxide component, CO was activated by the oxygen vacancy, while H_2_ was dissociatively absorbed on the Zn–O site. This resulted in an intermediate, CH_3_OH/DME, which was transferred to the CHA-type zeolite component and transformed to light olefins over the Brønsted acid sites, affording a C_2–4_^=^ selectivity of >74%, with a CO conversion of 10%–29% [99,100]. With ZnO-ZrO_2_/SSZ-13 as the catalyst, Wang *et al.* investigated the effect of Brønsted acid sites on product distribution. With a low density of Brønsted acid sites, the main product was CH_3_OH/DME, while an increase in the density of Brønsted acid sites resulted in the main product of light olefins [100]. Another key parameter was the proximity between the two components in the bifunctional catalyst. The close proximity between ZnO-ZrO_2_ and SAPO-34 favored the fast transfer of the intermediates (Fig. 6) [99]. However, increasing the proximity increased the risks of secondary hydrogenation and decreased selectivity to the light olefins. With ZnCrO_x_ as the oxide component, C_2–4_^=^ selectivity was enhanced to 80%, but the intermediate was proposed to be ketene because CH_4_ was the main product when only ZnCrO_x_ was used as the catalyst without a zeolite component [101]. The AEI-type SAPO-18 zeolite with similar pore sizes to CHA has been combined with ZnCrO_x_ and was used to catalyze the transformation of syngas to light olefins [102,103]. The C_2–4_^=^ selectivity reached 86.7% at a CO conversion of 25.2% [102], and low acid strength and density were revealed to favor a high olefin/paraffin ratio [102,103]. Thus, the use of another AEI-type zeolite, SSZ-39, as the zeolite component, with stronger acidity than that of SAPO-18, resulted in a high selectivity of 89% to liquefied petroleum gas (C_3_ and C_4_ paraffins) and particularly, a high propane selectivity of 80% at a CO conversion of 63% [104]. Notably, the delicate selectivity control in the light olefin range could be achieved using MOR as the zeolite component, cooperating with the ZnAl_2_O_4_ or ZnCrO_x_ metal oxide component, affording a high ethylene selectivity of 65% or 70%, respectively, although the reaction mechanism was different for the two distinct types of metal oxide components [105,106].

Aromatics can be synthesized from syngas in a two-stage process, that is, the conversion of syngas to methanol and the subsequent methanol to aromatics (MTA). With the aforementioned direct synthesis of light olefins from syngas via CH_3_OH/DME, the cooperation of metal oxide and the effective MTA catalyst ZSM-5, was applied to realize direct syngas to aromatics (STA). The bifunctional catalyst, ZnO-ZrO_2_/ZSM-5, afforded a high aromatic selectivity of 80% at 673 K under a syngas pressure of 3 MPa [107]. In contrast to the fast deactivation phenomenon ZSM-5 suffered during the MTA process [108,109], the bifunctional catalyst, ZnO-ZrO_2_/ZSM-5, was durable for at least 1000 h [107]. The optimal density of the Brønsted acid sites in the ZSM-5 zeolite differed for the MTA and STA processes. The density of the Brønsted acid sites was controlled at a low level for the syngas conversion, which prohibited coke deposition and resulted in higher stability than the MTA process. H_2_ atmosphere in the STA process also played a positive role in depressing coke formation. CO was confirmed to assist the formation of aromatics, as revealed by the isotopic labeling experiment during the MTA process [107]. Using ZnCrO_x_ as the metal oxide component, a slightly low aromatic selectivity (73.9%) was obtained at 623 K under a syngas pressure of 4 MPa [110]. Similarly, the Al amount should be controlled at a relatively low level, with the Si/Al ratio as high as 1066. With Ce-doped ZrO_2_ as the metal oxide component, the selectivity of aromatics increased to 83.1% at 653 K under a syngas pressure of 2 MPa [111].

Delicate selectivity control in the aromatic range was achieved by modifying the ZSM-5 zeolite. By removing the external acid sites of the ZSM-5 zeolite with tetraethoxysilane, the proportion of benzene, toluene and xylene increased from 30% to 65% in aromatics [107]. A core–shell structured zeolite component, with Zn-modified ZSM-5 as the core and all-silica S-1 as the shell, was adopted to cooperate with the ZnCrO_x_ component, resulting in an extremely high *para*-xylene selectivity of 77.3% in the xylene isomers [112]. With the bifunctional catalyst of the *b*-axis-orientated ZSM-5 zeolite and ZnCrO_x_, the total aromatics selectivity was 83.3% under an H_2_-deficient syngas (H_2_/CO = 1) condition, with a high tetramethylbenzene selectivity of 70% in total hydrocarbons [113]. The unique phenomenon of the hydrocarbon pool vacancy, created by selective methanol formation and fast diffusion along the *b* axis, was proposed to be the reason for the high tetramethylbenzene selectivity.

Studies on the direct conversion of syngas into liquid fuels, light olefins and aromatics are abundant and intensive, but those on the synthesis of the C_2+_ oxygenates from syngas are few. Wang *et al.* reported the direct synthesis of methyl acetate (MA) via the relay catalysis strategy, with Cu–Zn–Al oxide/H-ZSM-5 and the MOR zeolite as the catalyst, separated by quartz wool at ∼473 K [106]. The DME intermediate was formed over the Cu–Zn–Al oxide/H-ZSM-5 component, where H-ZSM-5 was responsible for the dehydration of methanol. Subsequently, the carbonylation of DME to MA and acetic acid (AA) occurred over the MOR component. At a high reaction temperature range of 603–643 K, the combination of ZnAl_2_O_4_ and the MOR zeolite afforded a long lifetime of ∼70 h, and the MA and AA selectivity was as high as >85% at a CO conversion of 11% [106]. Based on the intermediates of DME and MA, ethanol could be further produced as the main product by the combination of ZnAl_2_O_4_ and H-MOR in the form of ZnAl_2_O_4_|H-MOR|ZnAl_2_O_4_, affording the maximum ethanol selectivity of 64%.

CO_2_ could be transformed to CO via the reverse water–gas shift reaction; therefore, a similar bifunctional catalyst in the aforementioned processes starting from syngas can be applied in the hydrogenation of CO_2_ to liquid fuels, light olefins and aromatics. For example, the bifunctional catalyst, Na-Fe_3_O_4_/H-ZSM-5, yielded the main product of C_5–11_ at a selectivity of 78% at a CO_2_ conversion of 22% [114]. The direct synthesis of light olefins from CO_2_ can be achieved using ZnO-ZrO_2_/SAPO-34 at a high selectivity of 80% [115]. The ZnAlO_X_/H-ZSM-5 zeolite catalyzed the CO_2_ hydrogenation into aromatics at a high selectivity of 73.9% [116].

Notably, the aforementioned selectivities were calculated regardless of the CO_2_ in the syngas conversion and the CO in the CO_2_ hydrogenation. An effective catalyst is still desired to depress the water–gas shift reaction in the syngas conversion, and a reverse water–gas shift reaction occurred during the CO_2_ hydrogenation process.

## SYNTHESIS AND CATALYTIC APPLICATIONS OF METAL@ZEOLITE CATALYSTS

**Figure 7.**
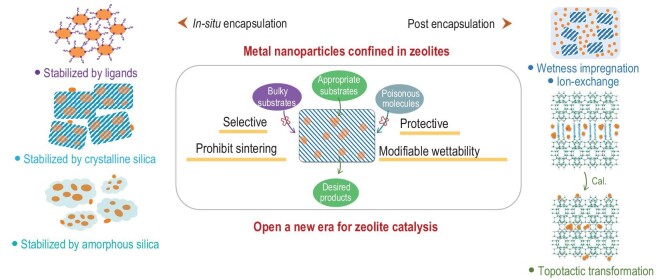
Synthesis of zeolite-confined metal catalysts with sintering-resistant properties.

In addition to their use as heterogeneous catalysts, zeolite materials can serve as excellent porous supports for metal or metal oxide to achieve high dispersion and unique confinement on a molecular scale (Fig. 7). Wet impregnation and ion exchange are the most universal methods to support metal active sites onto zeolite crystals regardless of the structure topology. However, the microporosity of zeolite supports imposes severe diffusion constraints for metal precursors, resulting in the non-uniform distribution of metal active sites and main location on the zeolite external surface. Owing to the relatively weak host–guest interaction, the metal nanoparticles on the zeolite surface suffer from severe sintering and deactivation at a high temperature. Kegnæs *et al.* introduced numerous intracrystal voids and mesopores into the framework of the S-1 zeolite via recrystallization and confined the Au nanoparticles mostly inside the S-1 zeolite with the impregnation method [117]. Similarly, Xiao *et al.* proposed a fish-in-hole strategy to encage Pd nanoparticles inside the mesopore traps of aluminosilicate zeolites via impregnation and constructed sinter-resistant catalysts for a CO oxidation reaction [118]. Even with mesopores, the full encapsulation of metal nanoparticles inside the zeolite matrix via impregnation was still difficult. To achieve full encapsulation, Ding *et al.* coated a silicate MFI layer over the micro/mesoporous ZSM-5 zeolite after the wetness impregnation of Pt species via dry–gel conversion [119].

When compared with the postencapsulation methods of impregnation and ion exchange, the simultaneous encapsulation of metal nanoparticles and the construction of zeolite frameworks are more attractive. In the past five years, a metal@zeolite ‘rush’ has been promoted with many studies reporting stable and active nano or even subnano metal particles confined in the zeolite matrix via *in-situ* hydrothermal synthesis or structural topological transformation [13,14,120]. For hydrothermal synthesis, the most important issue is to prohibit the precipitation of metal cations in an alkaline synthetic medium. Using organic ligands to stabilize metal cations is effective, with metal-N and metal-S complexes being the most frequently reported precursors. Iglesia *et al.* encapsulated noble metals, including Pt, Pd, Ru and Rh, into the intracrystal voids of small-pore zeolites (SOD, GIS, ANA and LTA) via direct hydrothermal synthesis, using ammonium or ethylenediamine as the ligand [121,122]. With the same ethylenediamine ligand, Yu *et al.* confined Pt clusters (∼1.8 nm) inside the pore walls of an S-1 zeolite via direct hydrothermal synthesis [123], serving as a highly stable and active catalyst for the dehydrogenation of formic acid. To increase the catalytic activity, Ni metal stabilized by the same ligands was encapsulated with Pd to form hybrid Pd-Ni(OH)_2_ clusters inside MFI crystals and increased the electron density on the Pd surface [124]. The metal-S complexes with mercaptosilane as the ligand were more powerful because the –SH groups could stabilize metal cations, and alkoxysilane groups could assist the crystallization of zeolite structures as a special silica source. Iglesia *et al.* introduced a variety of noble metals into the matrixes of LTA and MFI zeolites using 3-mercaptopropyltrimethoxysilane as the ligand [125,126]. Corma *et al.* adopted the same ligand to stabilize Pt cations in the synthetic gel of CHA zeolite, yielding tailorable Pt structures between atomic Pt species and subnano clusters (∼1 nm) [127].

The metal species could also be stabilized using an amorphous or crystalline silica matrix during hydrothermal synthesis. Christensen *et al.* immobilized Au nanoparticles in an amorphous matrix and used it as a silica source to synthesize S-1 zeolite, yielding 1–2 nm Au nanoparticles embedded in S-1 crystals [128]. However, the aggregation of Au on the surface of the S-1 zeolite was inevitable due to the mobility of metal species during hydrothermal synthesis. Xiao *et al.* reported a solvent-free synthetic strategy to reduce the mobility of metal species in amorphous silica and confined Pd or AuPd alloy nanoparticles in MFI crystals [88,129]. Su *et al.* stabilized Pt cations with Schiff silica via the charge electrostatic interaction between Pt and N atoms, and the Pt-containing silica source was crystallized to an MFI zeolite via dry–gel transformation. This prevented the problem of mobility in traditional hydrothermal synthesis [130]. Tang *et al.* constructed a series of yolk–shell zeolite-confined metal catalysts by stabilizing the metal species with the ammonium-modified mesoporous silica spheres, which were transformed into a crystalline S-1 shell with the assistance of seeds [131]. Using the seed-assisted method, Xiao *et al.* synthesized zeolite-confined metal catalysts by first encapsulating the metal nanoparticles inside the matrix of zeolite seeds and promoting zeolite crystallization on the surface of seeds with a core–shell growth mechanism, favoring the concentration of metal nanoparticles in the inner seed region [132,133]. With the interzeolite transformation strategy, the metal clusters pre-encapsulated in the parent crystalline zeolites of ^*^BEA or FAU can be encaged in the MFI zeolite [134].

Metal encapsulation can be achieved in the poststructural modifications of crystalline zeolites, such as the topotactic transformation and dissolution–recrystallization processes. Corma *et al.* introduced subnano Pt clusters into the swelling process of the MWW zeolite and encaged the Pt clusters inside the cups and pore walls around the cups, as well as the intracrystal supercages, during the topotactic transformation from a 2D swollen structure into a 3D structure upon calcination [135]. Assisted by a similar structural transformation, Zhang *et al.* introduced Pd cations into a swollen FER zeolite via ion exchange with surfactant cations, and encapsulated the Pd nanoparticles inside the 3D FER zeolite upon calcination [136]. The alkaline treatment of the S-1 zeolite resulted in a unique hollow nanobox morphology following the dissolution and recrystallization mechanism. Using this structural modification process and the pre-encapsulation of metals in an S-1 parent zeolite by wetness impregnation, certain studies have encaged a variety of mono- or bimetallic nanoparticles inside the S-1 hollow structure [137–140]. The size of the metal nanoparticles can be controlled by tailoring the concentration of metal precursors in the pre-impregnation process [141].

**Figure 8.**
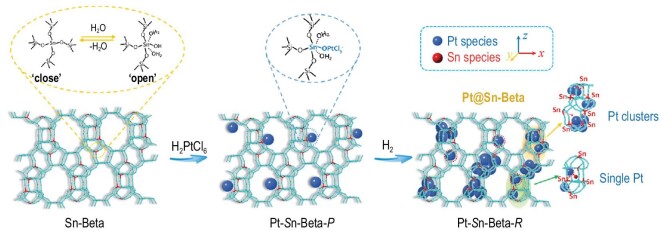
Framework heteroatom Sn as an anchor for subnano Pt confined in Beta zeolite. Reprinted with permission from ref. [143].

The heterogeneous atoms in the zeolite framework can serve as anchors for metal precursors to form isolated metal precursors and favor the formation of subnano metal clusters. Several reports have revealed the unique anchor effect of framework Sn species for Pt metals in direct synthesis or post-impregnation processes [142–145]. Wu *et al.* proposed the interaction between framework Sn species and Pt cations in the form of (SiO)_3_(H_2_O)_2_Sn(IV)-(O)-[PtCl_5_]^−^ groups (Fig. 8) [143]. In addition, the Sn species could enhance the activity and stability of Pt species in the dehydrogenation of propane to produce value-added propylene by modifying the electron structure of Pt active sites.

Metal@zeolite catalysts have shown great potential in numerous reactions and broken the bottleneck of certain industrial processes, such as CO oxidation, methane reformation, methane selective oxidation, selective hydrogenation and alkane dehydrogenation, owing to their high stability and unique selectivity. Despite being encapsulated inside zeolite crystals, metal active sites are still accessible to substrates through micropore channels [146]. As previously mentioned, the metal nanoparticles can be as small as single atoms or subnanometric clusters, stabilized by the nanospaces in zeolite structures. The smaller metal clusters with more unsaturated coordinated sites are considerably more active than the bulky clusters. By introducing KOH into the synthetic gels of silicate-1 confined Pd metal catalysts, Yu *et al.* controlled Pd clusters in the range of 0.3–0.6 nm, which was smaller than the Pd/C catalyst (∼3.8 nm) [123]. The ultrasmall Pd size and basicity created by K^+^ resulted in a ∼6-fold improvement in turnover frequency values, compared with Pd/C in the dehydrogenation reaction of formic acid, rendering it a potential catalytic system for H_2_ supply in small proton-exchange-membrane fuel-cell devices [123]. With the confinement of the zeolite structure, these highly active ultrasmall metal nanoparticles can resist sintering even under harsh conditions. During the high-temperature oxidation/reduction treatment under a similar condition used in industry, Corma *et al.* observed that the Pt confined in a CHA zeolite remained stable with a size of ∼1.3 nm, while the non-encapsulated Pt particles supported on amorphous SiO_2_ suffered severe sintering with the size increasing from ∼2 to ∼7 nm [127]. In those industrial processes, the high stability of the metal@zeolite catalysts was also confirmed. In the propane dehydrogenation process, the K-PtSn@MFI sample obtained in a one-pot synthesis exhibited a significantly enhanced lifetime compared to a reference K-PtSn/MFI material synthesized by wetness impregnation [142]. Xiao *et al.* revealed that Pt, Pd or Rh nanoparticles confined in a Beta or S-1 zeolite obtained using the seed-assisted method were highly stable in the catalytic conversion processes of C1 molecules, including water–gas shift reaction, CO oxidation, CH_4_ oxidative reforming and CO_2_ hydrogenation [133].

A zeolite shell with a unique pore structure and tunable hydrophilicity/hydrophobicity can endow metal@zeolite catalysts with unique selective properties. By confining Pd nanoparticles inside a Beta zeolite, Xiao *et al.* improved 4-chloroaniline selectivity to >99% in the catalytic conversion of 4-nitrochlorobenzene, compared to traditional Pd-supported catalysts (70.9%–89.6%) [132]. The adsorbate-displacement experiments revealed that the Beta zeolite shell changed the steric arrangement of the adsorption of 4-nitrochlorobenzene on Pd active sites and imposed a stronger interaction between the Pd sites and nitro groups than between the Pd sites and the –Cl groups [132]. Ding *et al.* reported a highly selective Pt@MFI catalyst in the hydrogenation of 4-nitrostyrene to produce 4-aminostyrene, while the reference Pt/MFI catalyst exhibited 100% selectivity for the undesired product of 4-ethylaniline [119]. The unique catalytic behaviors ascribed to the local environment of metal active sites imposed by the zeolite shell resembled enzyme catalysis. The shape selectivity was more general for the metal@zeolite catalysts, which confirmed the successful coating of metal by the zeolite shell [147]. In the alkene hydrogenation reaction, Corma *et al.* observed that the CHA-confined Pt nanoparticles converted 80% ethylene but only 20% propylene under identical reaction conditions because of the fast diffusion of small ethylene in the small-pore CHA zeolite [127]. Owing to the faster diffusion rate of furan in the S-1 zeolite than in other by-products, the Pd nanoparticles confined in an S-1 zeolite exhibited a remarkably higher furan selectivity of 98.7%, compared to that of the traditional Pd/S-1 catalyst (5.6%) in the hydrogenation of biomass-derived furfural [88]. The furan selectivity was enhanced to >99.9% at a full furfural conversion by increasing the hydrophilicity of the S-1 zeolite with additional silanol groups [148]. Similarly, by increasing the hydrophobicity of the ZSM-5 shell through silylation, the AuPd@ZSM-5 catalyst was highly active in the selective oxidation of methane to produce methanol, with the hydrophobic ZSM-5 shell serving as the molecular fence to concentrate *in-situ*-formed H_2_O_2_ [129]. The micropore channels in the zeolite shell can protect the metal active sites from being poisoned by bulky organic sulfides [126].

Although the metal clusters confined in the zeolite matrix can currently be controlled at the subnano or even atomic scale, a determination of precise structure, as well as a deep understanding of interaction mechanisms between metal and pore structures and heterogeneous atoms in the zeolite framework in catalytic reactions, are still required.

## CONCLUSION

Remarkable progress has been made in the synthesis and catalytic application of zeolite materials. Effective and green synthetic strategies have been developed, including the rational construction of zeolite structures via the fabrication of zeolitic monolayers, fast synthesis with the assistance of hydroxyl radicals, and green solvent-free synthesis. Bi/multifunctional and relay catalysis are realized by the combination of metal/metal oxide and zeolite, succeeding in the conversion of CO and CO_2_ to hydrocarbons with precisely controlled product distributions. Using zeolite as a porous shell to confine metal clusters increases the dispersion and stability of metal species and provides a confined reaction space with tunable pore sizes and hydrophilicity/hydrophobicity to achieve protective and selective catalysis. The delicate and rational control of zeolite synthesis and catalysis is a new development trend in zeolite science, and the mutual promotion between synthesis and catalysis will ensure continual updates in zeolite science.
